# Parathyroid Hormone in the Management of Pelvic Fragility Fractures: A Systematic Review and Meta-Analysis

**DOI:** 10.3390/jcm15031199

**Published:** 2026-02-03

**Authors:** Sophie A. Crooks, Kenan Kuršumović, Thomas L. Lewis, Nikolaos K. Kanakaris

**Affiliations:** 1 Royal Stoke University Hospital, Stoke-on-Trent ST4 6QG, UK; sophie.crooks@nhs.net (S.A.C.); kenan.kursumovic@uhnm.nhs.uk (K.K.); 2 Royal National Orthopaedic Hospital, Stanmore HA7 4LP, UK; 3 Leeds Major Trauma Centre, Academic Department of Trauma & Orthopaedics, University of Leeds, Leeds LS2 9JT, UK

**Keywords:** parathyroid hormone, teriparatide, pelvic fragility fractures, sacral fractures, osteoporosis, fracture healing, pain management, systematic review, meta-analysis

## Abstract

**Background**: Fragility fractures of the pelvis (FFPs) are increasingly prevalent given ageing populations. Conservative management is often primarily utilised due to its initial minimal displacement and the high risks of surgery in this vulnerable population. However, this can lead to rapid deconditioning, especially with non-weight-bearing protocols. Parathyroid hormone (PTH), as a bone anabolic agent, has the potential to improve clinical and radiological outcomes in FFPs, but the evidence remains limited. **Methods**: A systematic review and meta-analysis following PRISMA guidelines was undertaken. Database search results were independently screened by two authors, and data were extracted. The primary outcome measure was time to fracture healing as assessed by imaging, with the secondary outcome measure of pain levels (VAS/NRS). **Results**: There were 1230 articles screened, and 893 unique results identified. Six studies were included in the final analysis. These compared the use of PTH and its analogues with standard care, placebo, or sacroplasty. The findings suggest that PTH may accelerate fracture healing and reduce pain in this patient population, although evidence is limited and at high risk of bias. **Conclusions**: Treatment with PTH may improve bone healing and visual analogue pain scores, although the evidence is limited. There may be a benefit from adjunctive PTH treatment for patients with FFPs; however, larger methodologically robust studies are required to confirm this.

## 1. Introduction

**Figure 1:**
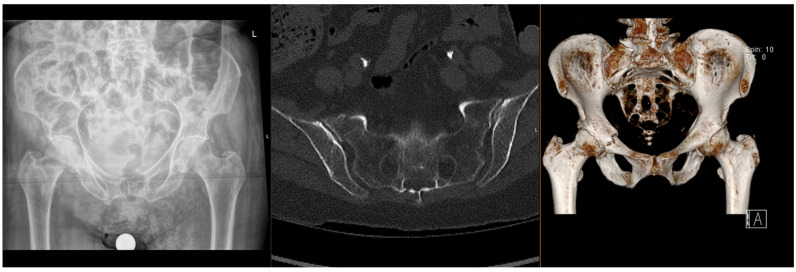
Radiographs and CT images demonstrating common FFP: lateral compression type.

**Figure 2:**
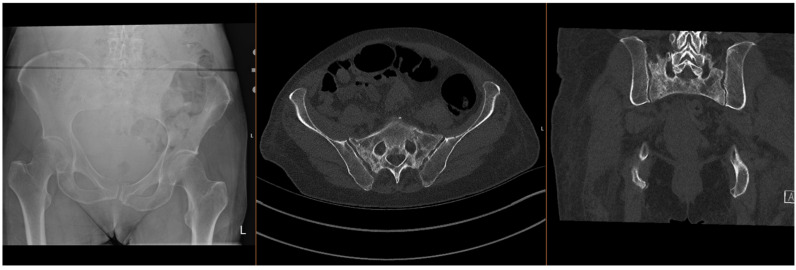
Radiographs and CT images demonstrating common FFP: comminuted bilateral sacral fractures.

Fragility fractures of the pelvis (FFPs) are increasingly prevalent worldwide, given the ageing population and increasing incidence of osteoporosis, which predisposes patients to sustaining these injuries with only minor trauma, or with physiological load in the absence of known trauma [1,2,3]. In the latter case, they are often referred to as insufficiency fractures, typically of the sacrum. Patients who sustain these fractures often have significant co-morbidities, which significantly increase the risks of surgical intervention [4,5]. The majority of FFPs (Figure 1 and Figure 2) are managed conservatively with analgesia and progressive mobilisation [6], but they also confer a significant health and social care burden and increased mortality risk [7,8], particularly if weight-bearing is restricted. Surgical treatment is reserved for displaced FFPs, those with clear features of mechanical instability, or following failure of non-operative treatment (primarily due to pain) [9,10,11,12].

There are high costs of prolonged rehabilitation and treatment for other medical issues secondary to immobilisation, such as hospital-acquired infections, failure to return to pre-injury mobilisation status, and often a requirement for long-term care input [13,14]. Any safe, non-surgical intervention that could improve outcomes may offer benefits not only at the individual patient level but also at the socioeconomic level [10,15,16,17].

Parathyroid hormone (PTH) and its analogues offer a promising option as a pharmaceutical adjunct for the treatment of fragility fractures in patients where early mobilisation is key to rehabilitation, but often difficult due to fracture-related pain [18]. PTH is secreted by the chief cells of the parathyroid gland and plays an important role in calcium homeostasis. When administered intermittently, PTH acts directly on osteoblasts to reduce osteoblast apoptosis, promote osteoblastogenesis, and reactivate dormant bone lining cells, thereby exerting an anabolic effect on bone [19,20,21,22,23].

Several parathyroid hormone analogues are available, including PTH 1-34 (teriparatide) and PTH 1-84. Teriparatide consists of the first 34 amino acids, which are the “active” part of the peptide, whilst PTH 1-84 includes the remaining “inactive” amino acids, with both formulations retaining the full biologic activity of the peptide. The efficacy of PTH treatment has been proven in both animal and human models, with an acceptable safety profile [24,25]. Peichl et al. showed that a once-daily injection of PTH 1-84 in patients with fragility fractures of the pelvis significantly accelerated time to union and overall union rates, as well as improving pain scores [26], and Lou et al. showed that teriparatide was effective in both accelerating fracture healing and improving functional outcomes in patients with osteoporotic fractures [27]. However, despite these potential benefits, the use of PTH is not widespread at present, perhaps due to the overall lack of clinical evidence and the additional cost in an already stretched health service.

This systematic review and meta-analysis aims to synthesise the evidence on PTH treatment for the management of FFPs and identify areas for future research.

## 2. Materials and Methods

A systematic review following the Preferred Reporting Items for Systematic Reviews (PRISMA) guidelines was undertaken [28], with the PRISMA checklist being available in the Supplementary Material. The protocol for this study was registered on the International Prospective Register of Systematic Reviews (PROSPERO) with the reference number CRD420251129699 [29].

### 2.1. Eligibility Criteria

Comparative or observational studies reviewing the use of PTH analogues in the treatment of patients with acute fragility-related pelvic fractures, with details of treatment and outcomes, were included. Studies which combined pelvic fractures with other fracture types, such as neck of femur fractures, were excluded in order to focus purely on pelvic fragility fractures. FFPs described in these papers occurred with minimal or no trauma, affecting the posterior and/or anterior pelvic ring, as previously described in the Rommens and Hofmann classification [30]. Case reports, biomechanical studies, in vitro studies, and studies that did not specify clinical or radiographic outcomes were excluded.

### 2.2. Search Strategy

An initial exploratory search was conducted to identify relevant keywords for the search strategy. The search string (“teriparatide” OR “parathyroid hormone” OR “PTH” OR “rhPTH” OR “Forteo” OR “Forsteo”) AND (“fracture”) AND (“pelvi*” OR “acetabul*” OR “sacral” OR “sacrum” OR “pubic”) was used to search the PubMed, EMBASE, MEDLINE, and Cochrane databases, from inception until August 2025.

### 2.3. Selection Process and Data Extraction

The Rayyan Systematic Review Management Platform (Rayyan Systems Inc., Cambridge, MA, USA) was used to systematically review the search results, and the titles and abstracts of all articles were independently reviewed by two authors (SAC and KK). Each full text was reviewed for inclusion, and citations were screened to identify any additional studies; any discrepancies were discussed with the senior author (NKK). To minimise bias, the data were blindly extracted by two authors (SAC and TLL) into a standardised spreadsheet.

### 2.4. Outcome Measures

The primary outcome measure was time to fracture healing. The secondary outcome measure was pain level at the latest follow-up. Study characteristics were extracted, including study design, period, country, number of patients, age, sex, diagnosis, and PTH therapy protocol. Study outcomes extracted included mean follow-up, VAS pain scores, radiological evidence of healing, and reported complications.

### 2.5. Risk of Bias and Quality Assessment

The studies included in the analysis were assessed for methodological bias. Randomised control trials were evaluated using the RoB2 tool (revised Cochrane risk-of-bias tool for randomised trials) [31]. Non-randomised trials were assessed using the ROBINS-I tool (risk of bias in non-randomised studies of interventions) [32]. The Joanna Briggs Critical Appraisal Checklist was used to assess case series [33]. The studies were evaluated independently by two authors (SAC and TLL), and any disagreements were resolved by discussion with the senior author (NKK).

### 2.6. Statistical Analysis

All data analysis was conducted using RStudio (version 2025.09.1). For studies that could not be pooled, descriptive statistics were reported, including means, standard deviations, and ranges where available. A meta-analysis was performed on VAS pain scores at 8–12 weeks post-intervention. The inverse variance method was employed with outcomes expressed as mean differences (MDs) and 95% confidence intervals (CIs). Both common effect and random effects models were fitted. Heterogeneity was assessed using the Q statistic (significance test), I^2^ statistic (proportion of variation due to heterogeneity), and H statistic. Forest plots visualised individual study effects and pooled estimates. Statistical significance was set at *p* < 0.05.

### 2.7. Funding and Ethical Approval

There was no specific funding to support this study. Ethical approval was not required. None of the authors reports any conflicts of interest.

## 3. Results

**Figure 3:**
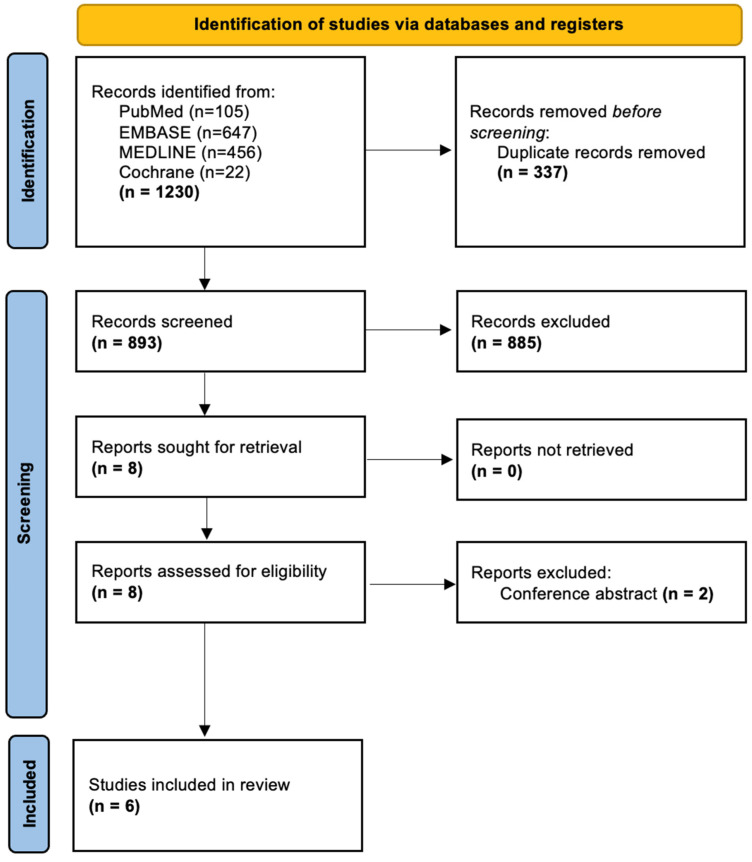
PRISMA flowchart demonstrating study inclusion.

The search process is shown in the PRISMA flowchart (Figure 3). A combined search of the PubMed, EMBASE, MEDLINE, and Cochrane databases yielded 1230 articles; after eliminating duplicates, 893 remained.

Following screening, a total of six studies were included in the final analysis [26,34,35,36,37,38]. These studies were published between 2011 and 2023. They included a total of 188 patients, with 86 treated with PTH [26,34,35,36,37,38] and the remaining 102 treated either with standard care (73) [35,38], placebo (15) [36], or sacroplasty/internal fixation (14) [35,37].

### 3.1. Study Characteristics

The characteristic features of all analysed studies are shown in Table 1 below:

### 3.2. Clinical Outcomes

Clinical and radiological outcomes are shown in Table 2 below:

Three studies, with a total of 56 patients in PTH groups and 77 in control groups, reported VAS pain scores at 8 to 12 weeks post-intervention, enabling quantitative synthesis [26,37,38]. Yang et al. [37] assessed outcomes at 12 weeks comparing PTH with sacroplasty, whilst Yoo et al. [38] and Peichl et al. [26] evaluated outcomes at 8 weeks comparing PTH with conservative management.

**Figure 4:**
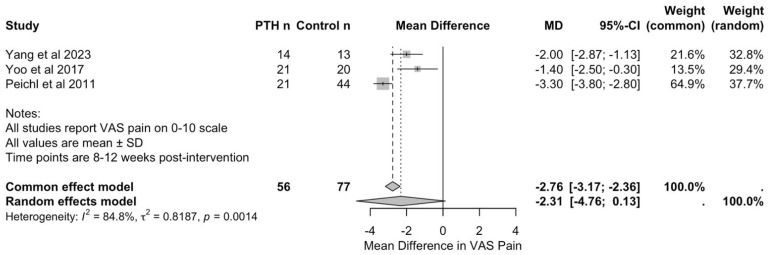
Forest plot of visual analogue scale for pain scores at 8–12 weeks following PTH therapy [26,37,38].

As seen in Figure 4, meta-analysis using the inverse variance method demonstrated a pooled mean difference of −2.31 (95% CI: −4.76 to 0.13) in favour of PTH. However, this did not reach statistical significance (*p* = 0.0553). The common effect model yielded a mean difference of −2.76 (95% CI: −3.17 to −2.36, *p* < 0.001); however, this should be interpreted with caution, given the substantial heterogeneity of the studies.

Heterogeneity analysis revealed considerable variation between studies (I^2^ = 84.8%, τ^2^ = 0.82, Q = 13.17, and *p* = 0.0014). This heterogeneity likely reflects differences in PTH dosing protocols (20 micrograms daily in Yang et al.’s [37] and Yoo et al.’s [38] studies versus 100 micrograms daily in Peichl et al.’s study [26]), in treatment duration (3 to 24 months), in control interventions (sacroplasty versus conservative management), and in the timings of outcome assessment (8 versus 12 weeks).

Individual study results consistently favoured PTH treatment. Yang et al. [37] reported VAS pain scores of 1.8 (SD: 0.6) in the PTH group compared with 3.8 (SD: 1.5) in the sacroplasty group at 12 weeks. Yoo et al. [38] reported scores of 3.4 (SD: 1.8) versus 4.8 (SD: 1.8) in the conservative group at 8 weeks. Peichl et al. [26] demonstrated the largest treatment effect, with scores of 3.2 (SD: 1.0) in the PTH group versus 6.5 (SD: 0.9) in the conservative group at 8 weeks. Leave-one-out sensitivity analysis confirmed the stability of the overall findings, with no single study exerting undue influence on the pooled estimate. Given the small number of included studies (n = 3), formal assessment of publication bias was not meaningful and is, therefore, not reported.

### 3.3. Complications and Adverse Events

Adverse effects were reported on by Yang et al. [37], Nieves et al. [36], and Peichl et al. [26], of which only Nieves reported any complications; there were eight serious adverse events (four in each group), none of which were believed to be related to the study medication [36]. The nature of the serious adverse events was not specified.

### 3.4. Risk of Bias and Quality Assessment

**Figure 5:**
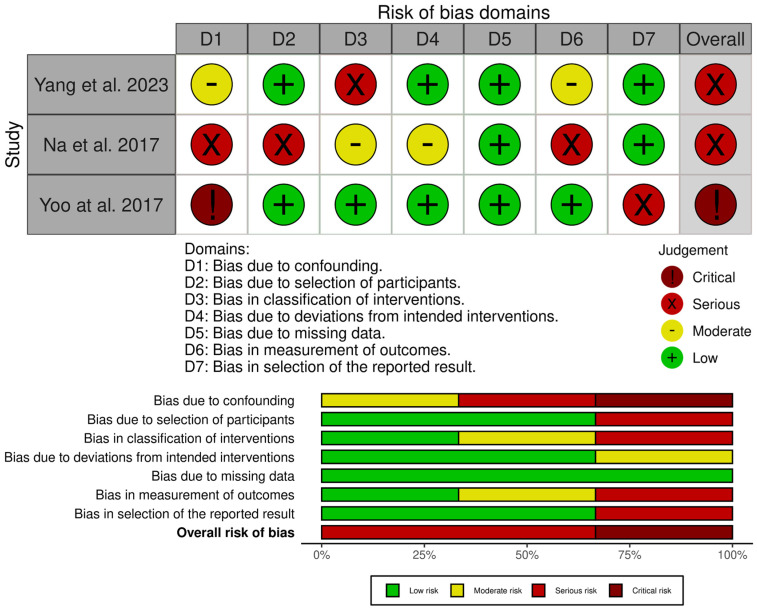
Risk of bias assessment of included studies—ROBINS-I tool [32,35,37,38,39].

**Figure 6:**
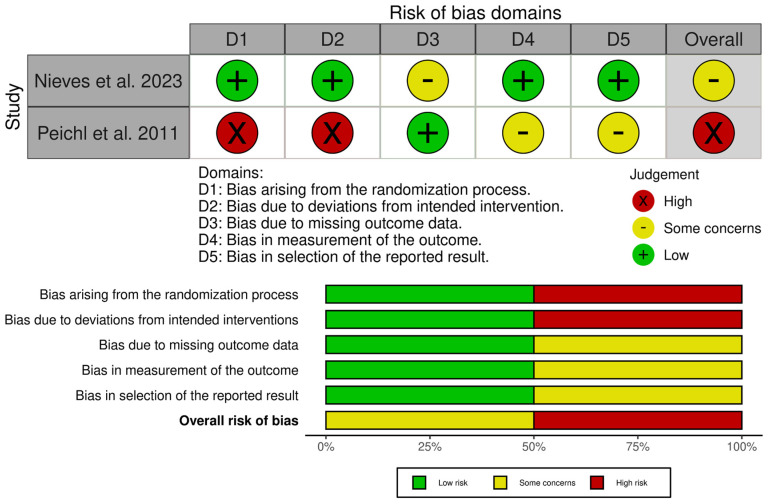
Risk of bias assessment of included studies—RoB 2 tool [31,26,36,39].

The included studies were assessed for their quality and risk of bias using the RoB 2 tool, the ROBINS-I tool (Figure 5 and Figure 6), and the JBI critical appraisal checklist for case series (for single-arm studies) [31,32,33]. Assessment across the domains (confounding, selection of participants, classification of interventions, deviations from intended interventions, missing data, measurement of outcomes, and selection of the reported result) indicated that none of the studies were judged to be at overall low risk of bias. Of the non-randomised comparative studies, all were at overall risk of serious or critical bias.

The JBI critical appraisal checklist was applied to the case series by Kasukawa et al. [34]. It was found to be of overall moderate quality, but small and uncontrolled, leading to a low level of evidence. Whilst the randomised study by Nieves et al. [36] was judged to be mostly of low concern, there was a degree of attrition imbalance between the groups, which led to an overall finding of some concern. However, sensitivity analyses did not change the results, and, therefore, it was felt that there was likely minimal bias toward overestimating the effect of PTH.

The randomised study by Peichl et al. [26] was found to be at high risk across multiple domains due to quasi-random allocation, lack of blinding of both patients and treating clinicians, and the fact that all intervention patients were treated at a single centre.

## 4. Discussion

This systematic review examined the efficacy of PTH in the treatment of FFPs, incorporating six studies reporting on 188 patients. The findings suggest that PTH may accelerate fracture healing and reduce pain in this patient population. However, the quality of the evidence remains limited and is at high risk of bias.

The two randomised controlled trials included in this review differed substantially in their design and outcomes. Peichl et al. [26] demonstrated superior radiographic healing rates and reduced pain scores at 8 weeks in patients receiving PTH compared with controls, whilst Nieves et al. [36] found no significant difference in CT-based union rates at 3 months. However, the latter study was terminated prematurely due to drug supply issues and was acknowledged by the authors to be underpowered, limiting the conclusions that can be drawn.

The four retrospective studies consistently reported improvements in pain scores. They showed reduced time to radiographic healing, with Na et al. [35] reporting a statistically significant shorter time to continuity of cortical bone seen on a CT scan in the PTH group (*p* = 0.012), with a time to union of 21.6 weeks in the PTH group, as opposed to 30.0 weeks in the control group. Yoo et al. [38] reported mean healing times of 7.8 weeks in the PTH group compared with 13.6 weeks in the control group. However, in the paper by Yoo et al. [38], there were significant baseline differences between the groups in BMI and vitamin D levels (with the control group having significantly lower BMI and Vitamin D levels at the time of admission). These were not accounted for in their analysis and, therefore, could provide a confounding effect in terms of both pre-fracture bone mineral density [40] and fracture healing [41,42], in addition to any impact of PTH supplementation. The time to mobilisation was also inconsistently reported in the paper, varying from 2.0 +/− 0.3 weeks to 4.0 +/− 0.3 weeks. Access to patient-level data could have enabled further analysis to clarify this aspect; however, this was not possible.

A key limitation of the existing literature is the substantial heterogeneity in PTH protocols employed. Dosing regimens ranged from 20 to 100 micrograms daily, with treatment durations from 3 to 24 months. This variation precludes direct comparison of outcomes across studies and makes it difficult to establish an optimal treatment protocol. The most common dose used was 20 micrograms daily, consistent with the approved regimen for osteoporosis treatment, although Peichl et al. [26] utilised 100 micrograms daily without reporting adverse events. This increased dose did not appear to be associated with any obvious change in outcomes from what was seen across all dosing regimens, and the necessity to give a higher dose than the accepted protocol for osteoporosis treatment is, therefore, not clear. However, it should be noted that the aims of treatment for osteoporosis and fragility fractures are different and, therefore, would benefit from further research with regards to determining the optimum dosing regimen specifically for FFPs.

The safety profile of PTH in this patient population appears favourable. Five of the six studies reported either no adverse events or adverse events that were not attributed to PTH therapy. Kasukawa et al. [34] noted that six of seven patients received concomitant medications, including non-steroidal anti-inflammatory drugs, vitamin supplements, and other bone-active agents, which may have influenced outcomes but reflect real-world clinical practice in the management of osteoporotic fractures.

VAS pain scores were the only outcome measure reported consistently across studies, enabling meta-analysis at the 8- to 12-week timepoint. All studies demonstrated reductions in pain scores following PTH treatment, suggesting that pain relief may be a more consistent and clinically relevant endpoint than radiographic healing, which varied in definition and assessment method across studies. The clinical significance of this finding is substantial, as pain control and functional recovery are primary concerns for elderly patients with FFPs.

Whilst a previous meta-analysis by Hong et al. [25] looking at the effect of PTH across all fracture types reported that the effectiveness and safety of PTH for fracture healing was reasonably well-established and credible, a subsequent meta-analysis by Eastman et al. [43] found that there was no evidence that PTH treatment improved fracture healing rates. Both meta-analyses found improvements in VAS pain scores in the PTH groups (Hong et al. [25], *p* = 0.06; Eastman et al. [43], *p* = 0.002); however, the effect did not reach the minimal clinically important difference (MCID) in either study [44]. In our meta-analysis, the difference did not reach statistical significance (*p* = 0.0553); however, the size of the difference would have exceeded the MCID, and further studies are, therefore, recommended to confirm or refute this. Eastman et al. [43] also reported that PTH treatment significantly improved functional outcomes for patients with fractures; however, when we specifically reviewed FFPs, functional outcomes were not reported consistently enough to allow for meta-analysis to be carried out.

The generalisability of the findings reported in this review is limited by several factors. All studies enrolled predominantly elderly female patients with established osteoporosis, and most explicitly focused on sacral fractures. The applicability to other pelvic fracture patterns, younger patients, or male populations remains uncertain. Furthermore, the retrospective nature of most included studies introduces inherent selection bias and limits the ability to establish causality. We note that whilst the majority of studies (four of six) were carried out in Asia, the number of patients was evenly split geographically between Asia (90 patients across Taiwan, Korea, and Japan) and Europe and North America (98 patients across the USA and Austria).

Future research should prioritise adequately powered randomised controlled trials with standardised PTH protocols and consistent outcome measures. Long-term follow-up is necessary to determine whether accelerated healing translates into sustained functional improvements and reduced complications such as non-union or chronic pain. Determination of optimal dose and cost-effectiveness analyses would also be valuable given the expense of PTH therapy [45]. Based on available data from the British National Formulary with regard to costings, the shortest and lowest dose protocol, as used by Nieves et al. [36] (PTH 1-34 20 μg/day for 3 months), would cost around £732.03 for the total treatment course [45], whereas the protocol used by Kasukawa et al. [34] (PTH 1-34 20 μg/day or 56.5 μg/week for 12 months) would cost around £2928.12 for the total treatment course [45] (the 56.5 μg/week formula is not available in the UK). Global production of rhPTH 1-84 was discontinued at the end of 2024, so the protocol used by Peichl et al. [26] (PTH 1-84 100 μg/day for 24 months) is no longer readily available [46].

## 5. Conclusions

In conclusion, whilst the available evidence suggests potential benefits of PTH in accelerating healing and reducing pain in pelvic fragility fractures, the quality of evidence remains moderate to low. The findings support consideration of PTH as an adjunct therapy in carefully selected patients, particularly those with significant pain or delayed healing, but further high-quality research is needed before definitive recommendations can be made. Given the potential benefits of early mobilisation, reduced pain and improved healing rates, the role of PTH therapy should be further explored.

## Figures and Tables

**Table 1:** Characteristics of studies included for review, showing country of origin and study design, number and sex of patients included, minimum follow-up, inclusion criteria, and PTH treatment protocol.

Study ID	Country	Patients (n=)	Sex	Follow-Up (Months)	Design	Inclusion Criteria	PTH Treatment Protocol
Yang et al., 2023 [37]	Taiwan	27	Female only	6	Retrospective observational study	All female patients diagnosed with sacral insufficiency fractures between 2011 and 2021	20 μg/day for 6 months
Nieves et al., 2022 [36]	USA	33	Both	12	Randomised parallel-design phase 2 study (double-blind)	All patients > 50 years; acute fractures with minimal trauma presenting within 1 month of symptoms	20 μg/day for 3 months
Na et al., 2017 [35]	Korea	15	Both	12–18	Retrospective observational study	Patients attending the hospital due to difficulty in ambulation caused by pelvic pain despite no or minor trauma between April 2011 and March 2014	20 μg/day “until fracture healing”
Kasukawa et al., 2017 [34]	Japan	7	Female only	6–24	Retrospective observational study	Patients with osteoporosis and sacral insufficiency fractures	20 μg/day (5 patients) OR 56.5 μg/week (2 patients) for 6–12 months
Yoo et al., 2017 [38]	Korea	41	Both	12	Retrospective case-controlled study	Consecutive patients > 50 years, no signs of infection, never had radiation therapy, available for follow-up >1 year, and confirmed sacral insufficiency fractures on MRI/bone scan	20 μg/day for 3–11 months
Peichl et al., 2011 [26]	Austria	65	Female only	24	Randomised controlled trial	Female patients, unilateral pelvic fracture, age > 70 years, and osteoporosis with T-score < −2.5	100 μg/day for 24 months

**Table 2:** Characteristics of studies included for review, showing the number of patients in each group, the nature of comparator groups, VAS at admission and final assessment, radiographic healing criteria and outcomes, and complications.

Study ID	Group (n=)	Admission VAS (cm)	Final VAS (cm)	Time at Final VAS	Radiographic Healing Criteria	Radiographic Healing Outcomes	Complications
Yang et al., 2023 [37]	PTH (14)	8.0 ± 1.0	0.6 ± 0.8	24 weeks	Presence of callus or sclerosis at the fracture site on AP and lateral radiographs at 12 weeks	6/14 callus 6/14 sclerosis	None
	Sacroplasty (13)	7.7 ± 0.8	2.7 ± 1.4			6/13 callus 1/13 sclerosis	
Nieves et al., 2022 [36]	PTH (18)	-	-	-	CT-modified version of the RUST scoring index at 12 weeks. “Healed” is defined as the bridging of 3 or 4 cortices	50% healed	8 adverse events (4 in each group) Not believed to be related to study medication
	Placebo (15)	-	-	-		53% healed	
Na et al., 2017 [35]	PTH (5)	7.2	3.2	Not stated	Continuity of cortical bone on CT scan	21.6 weeks to union (18–27)	Not stated
	Conservative (10)	7.44	3.67			30 weeks to union (22–34)	
Kasukawa et al., 2017 [34]	PTH (7)	8.74 ± 1.1	1.29 ± 1.49	24 weeks	Not stated	100% union or sclerosis seen on CT at 24 weeks	Not stated
Yoo et al., 2017 [38]	PTH (21)	6.9 ± 1.5	3.4 ± 1.8	8 weeks	Cortical bridging on plain radiograph at 8 weeks	100% healed	Not stated
	Control (20)	6.5 ± 2.5	4.8 ± 1.8			10% healed	
Peichl et al., 2011 [26]	PTH (21)	7.6 ± 1.1	3.2 ± 1.0	8 weeks	Cortical bridging on CT at 8 weeks	100% healed	None
	Control (44)	7.7 ± 1.1	6.5 ± 0.9			9.1% healed	

## Data Availability

No new data were created or analyzed in this study.

## Supplementary Materials

The following supporting information can be downloaded at: https://www.mdpi.com/article/10.3390/jcm15031199/s1, PRISMA checklist as per the PRISMA 2020 statement [28].

## Abbreviations

The following abbreviations were used in this manuscript:

PTH	Parathyroid hormone
FFP	Fragility fracture of the pelvis
VAS	Visual analogue scale
MCID	Minimum clinically important difference
